# Abernethy malformation (Type II) presenting in a 6-day-old boy with Noonan syndrome: a case report

**DOI:** 10.1186/s12887-025-05726-1

**Published:** 2025-07-12

**Authors:** Yujuan Wang, Wei Wang, Xiaoru Wang, Xiaowei Xin, Yi Yin, Chun Zhao, Hua Jin, Youpeng Jin

**Affiliations:** 1 Department of Pediatric intensive care unit, Shandong Provincial Hospital Affiliated to Shandong First Medical University, No. 324, Jingwu Road, Huaiyin District, Jinan, 250021 Shandong Province P. R. China; 2 Prenatal Diagnosis Center, Jinan Maternity and Child Care Hospital Affiliated to Shand ong First Medical University, Jinan, China; 3 Department of Pediatric intensive care unit, Cheeloo College of Medicine, Shandong Provincial Hospital, Shandong University, Jinnan, 250021 Shandong Province P. R. China; 4 Department of Pediatric Intensive Care Unit, Children’s Hospital of Chongqing Medical University, Chongqing, 400014 China

**Keywords:** Abernethy malformation, Noonan syndrome, Congenital extrahepatic portosystemic shunt, Pediatric, *LZTR1* gene

## Abstract

**Background:**

Abernethy malformation (AM) is a rare vascular anomaly characterized by the diversion of splanchnic venous blood directly into the systemic circulation, bypassing the liver. We present the clinical features, diagnostic workup, and follow-up of a 6-day-old Chinese male with type II AM combined with Noonan syndrome (NS).

**Case presentation:**

The patient was prenatally suspected of having AM based on ultrasonographic findings, which were postnatally confirmed through enhanced computed tomography (CT) and magnetic resonance (MR) imaging. Due to dysmorphic facial features, whole-exome sequencing (WES) was performed, identifying a heterozygous c.848G > A (p. Arg283Gln) variant in the *LZTR1* gene (NM_006767.4), consistent with NS. During follow-up, the patient exhibited progressive elevation of liver enzymes and hyperammonemia, prompting laparoscopic portosystemic shunt ligation at six months of age. Postoperatively, the patient demonstrated rapid biochemical normalization and sustained clinical improvement.

**Conclusions:**

In patients with RASopathies, clinicians should maintain a high index of suspicion for AM and NS. Comprehensive vascular evaluation, particularly imaging screening of the portal venous system, is essential to avoid missed diagnoses and ensure timely intervention, thereby improving patient outcomes. A multidisciplinary approach that integrates genetic testing, advanced imaging, and surgical expertise is essential for optimizing outcomes in these complex cases.

## Background

Abernethy malformation (AM) is sometimes referred to as congenital extrahepatic portosystemic shunt that is characterized by the drainage of portosystemic blood in such a way that bypasses the liver to directly enter systemic circulation. Initially described in 1793 by John Abernethy [1]. It is a very rare condition that often coincides with a range of other anomalous findings [2].

Notably, the coexistence of AM with Noonan syndrome (NS), which is an autosomal dominant RASopathy caused by pathogenic variants in the RAS-MAPK pathway genes, that can affect a range of organs throughout life [3], remains exceptionally rare.

Here, we report a novel case of a Chinese male neonate with Type II AM and NS harboring a heterozygous pathogenic variant in Leucine-zipper-like post translational regulator 1(*LZTR1)*. The patient successfully underwent laparoscopic portosystemic shunt ligation at six months of age following progressive elevation of hepatic transaminases and hyperammonemia, with subsequent normal growth, liver function, blood ammonia, coagulation function and neurodevelopmental progress postoperatively.

## Case presentation

A 6-day-old Chinese male neonate was admitted to the pediatric intensive care unit (PICU) of Shandong Provincial Hospital Affiliated to Shandong First Medical University with a 1-day history of tachycardia. He was the first child of a healthy, nonconsanguineous couple, delivered via cesarean section at 36 weeks and 5 days of a gestation following a pregnancy complicated by prenatal ultrasound findings suggestive of AM. Prenatal ultrasound examination at 27 weeks of gestation revealed tortuous dilatation of the extrahepatic portal vein, horizontal renal pole displacement across the abdominal aorta, and proximal inferior vena cava (IVC) dilatation. At birth, the infant exhibited normal APGAR scores (9 at 1 min, 10 at 5 min), with a birth weight of 3,620 g and length of 49 cm. Shortly after delivery, he developed paroxysmal tachycardia with a maximum heart rate of 280 bpm, prompting PICU admission.

On admission, this boy was in poor condition, manifested with dyspnea, tachycardia (280 bpm; normal 110–160), and peripheral cyanosis (Saturation of Peripheral Oxygen, 82% on room air). Physical examination revealed subcostal retractions, nasal flaring and dysmorphic features including high-arched palate, low-set ears, pectus excavatum and left cryptorchidism(untouched testis).Initial laboratory tests demonstrated abnormal coagulation parameters(prolonged prothrombin time[PT]:16.1 s[normal:10.7–14 s]; elevated international normalized ratio[INR]: 1.41[normal:0.8–1.2], hypoalbuminemia (serum albumin: 29.2 g/L [normal:40–55 g/L], and hyperammonemia (blood ammonia: 70µmol/L [normal:9–47µmol/L]. Routine echo cardiogram (ECG) confirmed atrial tachycardia. Echocardiographic examination revealed a patent foramen ovale and pulmonary arterial hypertension (PAH, pulmonary arterial systolic pressure, 46 mmHg). Chest radiography and lung ultrasound revealed no evidence of hyaline membrane disease, ground-glass opacities, or air bronchograms.

**Fig. 1:**
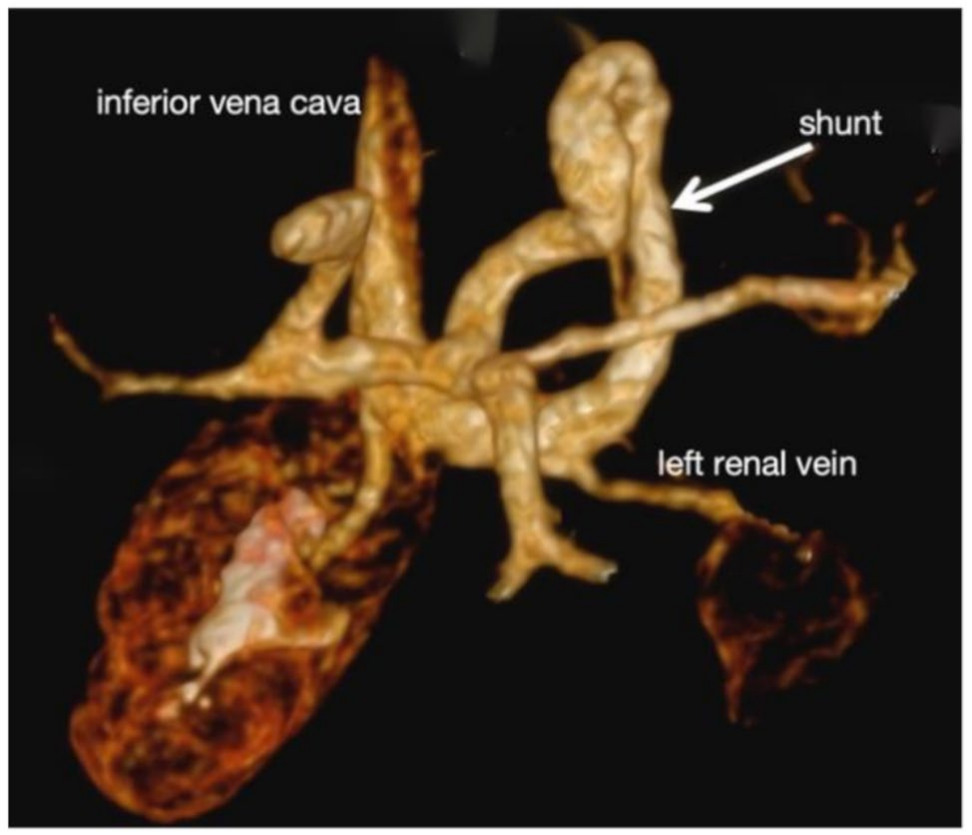
CTV of the inferior vena cava demonstrating dilatation of the left portal vein and its tributaries. An anomalous shunt vessel (white arrow) originated from the distal main portal vein. The shunt follows a tortuous course along the left hepatic margin, traverses the aortomesenteric angle (formed by the superior mesenteric artery and abdominal aorta), and terminates at the IVC near the left renal vein confluence

To confirm the suspected portosystemic shunt, portal vein magnetic resonance (MR) imaging and IVC computed tomography venography (CTV) were performed. Imaging revealed the thickening of the left portal vein, and a 7.1 mm shunt vessel originating from the distal main portal vein. The shunt coursed along the left hepatic margin, traversed the gastrosplenic region, passed through the superior mesenteric artery-abdominal aorta angle, and terminated at the left renal vein-IVC confluence (Fig. 1). These findings were diagnostic of Type II AM.

Given the patient’s dysmorphic facial features (high-arched palate, low-set ears) and skeletal abnormalities (pectus excavatum), whole-exome sequencing (WES) was performed to investigate potential genetic etiologies. WES identified a heterozygous c.848G > A (p. Arg283Gln) variant in the *Leucine-zipper-like post-translational regulator 1 (LZTR1)* gene (NM_006767.4), located at chr22:21345973 G > A (GRCh38). This variant has been previously reported in patients with Noonan syndrome (NS) and was classified as “Likely Pathogenic” according to the American College of Medical Genetics and Genomics (ACMG) guidelines (criteria: PS2 + PM1_P + PM2 + PP3) (Table 1). The parents of the proband did not carry this variant.

**Table 1:** Genetic testing results relevant to the clinical presentation of the proband

Gene	Variation location	HGVS	Variation type	Heterozygosity	variation rating	Disease and inheritance
*LZTR1*	chr 22-21345973	NM_006767.4 c.848G > A (p. Arg283Gln)	Missense	Heterozygous	Likely pathogenic	Noonan syndrome, AD

The patient was managed with high-flow nasal cannula (HFNC) oxygen therapy, intravenous amiodarone for rhythm control, and olprinone for pulmonary arterial pressure reduction. By day 23, his blood ammonia level had declined to 59 µmol/L, and he no longer required supplement oxygen. ECG confirmed normal sinus rhythm and echocardiographic confirmed normal pulmonary arterial pressure. Given the clinical stabilization, the patient was discharged without immediate surgical intervention.

At the 1-month post-discharge follow-up, the patient exhibited normal growth and development without recurrence of tachycardia or PAH. However, laboratory tests revealed elevated liver enzymes [Alanine aminotransferase: ALT, 73 U/L [normal: 9–50 U/L]; Aspartate aminotransferases: 113 U/L [normal: 15–40 U/L]) and persistent hyperammonemia (82 µmol/L, normal:9–47µmol/L). Coagulation parameters remained within normal limits (PT: 12.5 s; INR: 1.05). Viral hepatitis and other causes of liver injury were excluded through comprehensive serological testing. The patient was initiated on oral reduced glutathione as hepatoprotective therapy.

Despite these measures, progressive elevation of liver enzymes and ammonia levels was observed over the following months. By the 5-month follow-up, abnormal laboratory findings included ALT: 228 U/L, AST: 305 U/L, and blood ammonia: 104 µmol/L, accompanied by mild coagulopathy (PT: 14.9 s; INR: 1.33). These abnormalities were attributed to Type II AM. Consequently, the patient underwent laparoscopic portosystemic shunt ligation, which was performed without complications.

**Fig. 2:**
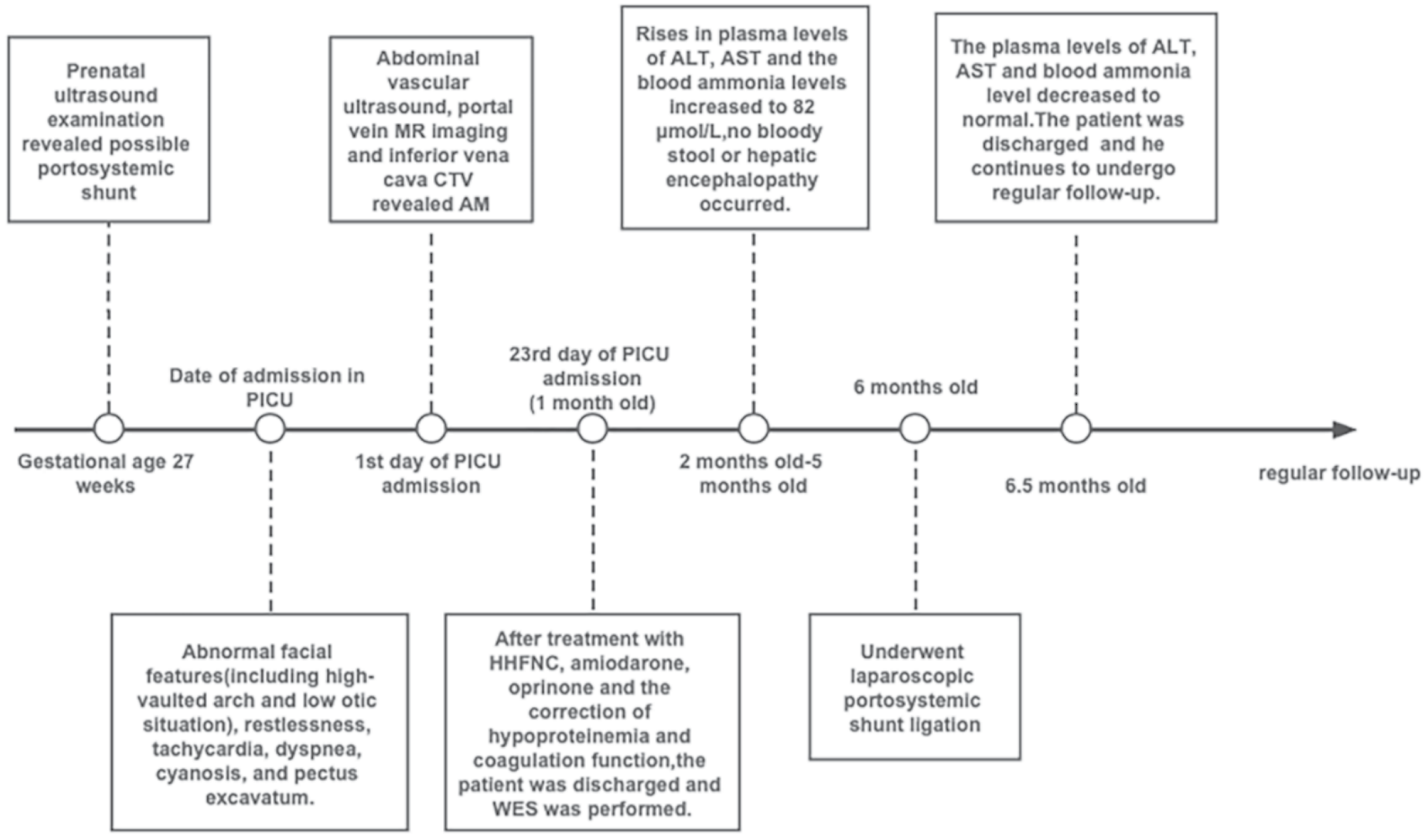
Timeline of patient’s clinical course

Postoperative recovery was uneventful. By postoperative day 5, liver enzymes and blood ammonia levels had normalized (ALT: 32 U/L; AST: 28 U/L; blood ammonia: 28 µmol/L) (Table 2). The patient was discharged on postoperative day 8 with a body weight of 7,500 g and length of 72 cm. Regular follow-up continues to monitor growth, liver function, coagulation function and neurodevelopmental progress (Fig. 2).

**Table 2:** Laboratory results of the patient

Age (month)	ALT (U/L)	AST (U/L)	Serum albumin (g/L)	Blood ammonia (µmol/L)	PT (second)	INR
0*	14	20	29.2	70	16.1	1.41
1*	18	27	36.6	59	12.6	1.12
2*	73	113	40.7	82	13.1	1.18
5*	137	113	39.6	61	12.4	1.11
6*	228	305	41	104	14.9	1.33
6.5^#^	29	38	39.3	29	11.3	1.1
8^#^	14	23	40.4	21	11.4	1.03
12^#^	7	21	41.2	20	11.5	1.04

ALT: Alanine aminotransferase (reference interval:9-50U/L); AST: Aspartate aminotransferase (reference interval:15-40U/L); PT: Prothrombin time(reference interval:10.7–14 s); INR: International normalized ratio [reference interval 0.8–1.2]; Serum albumin: reference interval 40–55 g/L; Blood ammonia: reference interval: 9–47µmol/L; * means pre-surgery values;# means post-surgery values

## Discussion

AM is a rare condition wherein the portomesenteric blood supply drains via a complete or partial shunt into the systemic blood supply, bypassing the liver. This condition affects roughly 1 of every 30,000–50,000 live births [4], and is categorized into two types based on the pattern of the portal and systemic vein patterns. According to current literature, Type I AM appear to exhibit a female predominance, while Type II AM demonstrates a higher prevalence in males [5, 6]. In type I cases, complete aplasia of the intrahepatic portal vein branches and complete extrahepatic shunting of portovenous blood into systemic veins is observed, often associated with congenital anomalies, and increased risk of hepatic tumors [5]. In type II cases, hypoplastic intrahepatic portal vein branches are observed together with the partial extrahepatic shunting of the portovenous blood into a systemic vein.

Clinical manifestations of AM patients may exhibit a wide array of clinical symptoms including hyperammonemia and jaundice [7], with the precise nature of these symptoms being dependent on portosystemic shut locations. This condition is most often diagnosed in children exhibiting congenital defects, psychomotor delay, cholestasis, failure to thrive, or hyperglycemia. In some instances, it may be detected incidentally via prenatal ultrasonography [8, 9] or during the evaluation of portopulmonary syndrome, abdominal pain, portopulmonary hypertension, abnormal liver function test results, or portosystemic encephalopathy. In the present case, the patient’s hyperammonemia and regressive elevation of liver enzymes likely resulted from the systemic circulation of unmetabolized ammonia due to the portosystemic shunt. Although the exact pathophysiology of pulmonary hypertension caused by congenital portosystemic shunts remains incompletely elucidated, proposed mechanisms include volume overload due to increased systemic blood flow and impaired hepatic clearance of vasoactive substances [10].

Ultrasound examination, enhanced CT, and MR imaging can all aid efforts to diagnose AM. Prenatal diagnosis, as demonstrated in our case, is increasingly feasible with ultrasonography [11]. Although Doi et al. emphasized angiography as a mandatory requirement for diagnosing AM [12], our case successfully confirmed the presence of a portosystemic shunt via CTV without evidence of portal hypertension on imaging. The absence of collateral vessels or splenomegaly further excluded significant portal hypertension, fulfilling the diagnostic benchmarks non-invasively.

The patient in the present case also presented with atrial tachycardia and dysmorphic facial features, which may be associated with his concomitant NS. NS, which is a type of RASopathy caused by dysregulation of the RAS/MAPK signaling pathway [13], is an uncommon autosomal dominant/ recessive condition that has been tied to variations of roughly 20 genes to date [14], affecting 1 in every 1,000–2,500 live births [15]. NS is one of the largest groups of multiple congenital anomaly disorders and is characterized by distinctive facial features, short stature, learning difficulties, delayed development, bleeding difficulties, lymphatic malformations, renal abnormalities, and congenital heart disease [16]. Patients with NS typically present cardiovascular anomalies, with pulmonary stenosis, hypertrophic cardiomyopathy, and atrial septal defects [17, 18]. Molecular genetic testing can provide confirmation for most NS patients.

The *LZTR1* gene, which encodes a Golgi-localized BTB-Kelch superfamily protein critical for RAS/MAPK pathway regulation [19], has been definitively linked to Noonan syndrome type 10 (NS10) through gain-of-function variants that drive constitutive pathway activation [20, 21]. Our identification of the *LZTR1*c.848G > A variant (ACMG-classified “Likely Pathogenic”) expands the molecular spectrum of NS-associated vascular pathology, with integrated analysis of published cases [11, 19, 20, 22–24]revealing two distinct clinical trajectories: coronary anomalies (type I AM featuring left coronary artery fistulae and anomalous origins in *LZTR1*-variant carriers) versus portosystemic shunting (type II AM associated with either *PTPN11* mutations [23] or genetically undefined cases [11, 24]). This phenotypic dichotomy, coupled with reports of lethal vascular complications (sudden cardiac death in a *LZTR1*-variant carrier with COVID-19 comorbidity [22]), strongly implicates RAS/MAPK hyperactivation in endothelial dysregulation and aberrant vasculogenesis.

These cases underscore the necessity of comprehensive vascular system evaluation in patients with RASopathies, including systematic imaging screening of the portal venous system, to minimize the risk of missed diagnoses. Multicenter collaborative efforts are needed to establish comprehensive genotype-phenotype correlations and elucidate whether AM represents a direct consequence of RASopathy or a coincidental comorbidity.

The management of AM is highly individualized, depending on the type of malformation, associated anomalies, and clinical complications. There is no universal therapeutic strategy, and treatment decisions must be tailored to each patient’s specific presentation. In general, patients with type I Abernathy malformation require liver transplantation, while shunt closure can be sufficient to treat type II cases [25–27]. Treatment is necessary for any patient with symptoms, and the shunt ratio, as computed via doppler ultrasonography, is used to guide treatment efforts for asymptomatic individuals. When the shunt fraction, defined by the ratio of the shunt flow to the total portal flow volume, exceeds 60%, patients face a high risk of hepatic encephalopathy such that treatment is generally advisable. Some expert opinions indicate that children diagnosed with AM should undergo endovascular intervention or surgical treatment as soon as possible in symptomatic cases [28]. The primary goals of treatment include reducing systemic ammonia levels, alleviating symptoms, and preventing long-term complications through the restoration of physiological portal flow [29]. Per current guidelines for management [18], most patients with NS achieve normal intellectual performance in adulthood, although they require routine follow-up to monitor for complications such as hypertrophic cardiomyopathy and lymphatic abnormalities.

In our patient, progressive elevation of liver enzymes and hyperammonemia during follow-up prompted laparoscopic portosystemic shunt ligation at six months of age. Postoperatively, the patient exhibited rapid normalization of biochemical parameters including the level of liver enzymes, blood ammonia and coagulation parameters, and remained stable at the 12-month follow-up, with no recurrence of symptoms. For NS, the patient continues to undergo regular multidisciplinary evaluations to monitor growth and neurodevelopment.

## Conclusion

This case underscores the diagnostic challenges associated with AM in Noonan syndrome. It emphasizes the necessity of comprehensive vascular evaluation in patients with RASopathies, particularly the imaging screening of the portal venous system, to prevent misdiagnosis and ensure timely intervention. Prenatal ultrasonography can facilitate the early detection of AM, thereby enabling timely postnatal management. Additionally, molecular genetic testing is crucial for confirming the diagnosis of NS. A multidisciplinary approach that integrates genetic testing, advanced imaging, and surgical expertise is essential for optimizing outcomes in these complex cases.

## Data Availability

The data in this article are deposited in the National Genomics Data Center(NGDC, https://ngdc.cncb.ac.cn/)repository, HRA006770.

## Abbreviations

AM: Abernethy malformation
NS: Noonan syndrome
*LZTR1*: *Leucine-zipper-like post translational regulator 1*
PICU: pediatric intensive care unit
IVC: inferior vena cava
PT: prolonged prothrombin time
INR: international normalized ratio
ECG: echo cardiogram
PAH: pulmonary arterial hypertension
MR: magnetic resonance
CTV: computed tomography venography
WES: whole-exome sequencing
ACMG: American College of Medical Genetics and Genomics
HFNC: high-flow nasal cannula
ALT: Alanine aminotransferase
AST: Aspartate aminotransferase
